# Eating Behavior during First-Year College Students, including Eating Disorders—RUVIC-RUNEAT-TCA Project. Protocol of an Observational Multicentric Study

**DOI:** 10.3390/ijerph18189457

**Published:** 2021-09-08

**Authors:** Anna Vila-Martí, Iñaki Elío, Sandra Sumalla-Cano

**Affiliations:** 1 Research Group M3O, Methodology, Methods, Models and Outcomes of Health and Social Sciences, Faculty of Health Sciences and Welfare, University of Vic-Central University of Catalonia, Vic 08500, Spain; 2 Research Group on Foods, Nutritional Biochemistry and Health, Universidad Europea del Atlántico, 39011 Santander, Spain; inaki.elio@uneatlantico.es (I.E.); sandra.sumalla@uneatlantico.es (S.S.-C.); 3 Department of Health, Nutrition and Sports, Campus Campeche, Universidad Internacional Iberoamericana, Campeche 24560, Mexico

**Keywords:** anorexia nervosa, bulimia nervosa, Adherence to the Mediterranean Diet, evolution of body composition, college students

## Abstract

(1) Introduction: Changes in eating behavior and eating disorders are especially common in young people, especially teenage and college women. The first year of college is a critical period, as students acquire freedoms that can lead to poor eating habits. During this first year, students usually gain weight. The aims of this project are to analyze the risk of developing eating disorders, the composition and dietary intake and the changes in the body composition of two groups of college students (independent from the family nucleus or still living within the family) in the first year of college. (2) Material and Methods: Multicentric prospective observational study protocol in which first-year students at the Universidad Europea del Atlántico and Universitat de Vic-Universitat Central de Catalunya voluntarily took part in the study. The students will be divided into two groups, independent and those residing in the family home, and the evolution of both groups will be compared at the beginning and at the end of the school year by performing anthropometric measurements, tests on lifestyle and eating habits (Test of Adherence to the Mediterranean Diet, MEDAS-14; Emotional Eater Questionnaire, EEQ), validated questionnaires on eating disorders (Eating Attitude Test, EAT26; Teen Figure Drawing Scales; SCOFF, Eating Behavior Test; Bulimia Investigatory Test Edinburgh, BITE) and their intake will be evaluated through 72 h dietary records. (3) Discussion: Determining the risk of suffering eating disorders of alimentary behavior, knowing eating consumption, perception of the corporal image and body composition through the first year of college will be decisive in establishing alimentary education strategies to prevent possible eating disorders in young students.

## 1. Introduction

For many students, starting college is not only a change in educational stage, but in many cases, these young adults are emancipated from their homes. This emancipation not only entails having to organize their studies but also their diet and lifestyle. Different studies have shown that food education in the previous stages of life is not enough for these young university students to know how to correctly develop good eating behavior [1,2]. Eating behavior not only refers to the ability to choose food, but also to culinary skills and the measures taken regarding food safety [3]. Therefore, this stage is the trigger for the appearance of unhealthy eating behaviors [4], such as eating disorders (EDs).

EDs are serious psychiatric illnesses linked to a distorted perception of one’s own body and bodily dissatisfaction and are characterized by marked alterations in behavior and excessive concern for weight and/or body shape [5]; they are especially common in young people.

The following are considered EDs: anorexia nervosa (AN), bulimia nervosa (BN), binge eating disorder (BED), unspecified eating disorder and obesity, and have recently been incorporated into EDs [6]. Ninety-five percent of eating disorders occur in women and are more common in high school and college students [7]. In addition, there is a strong relationship between anorexia and bulimia nervosa, and both occur mostly in healthy young women who begin to worry excessively about weight and physique. Approximately 50% of anorexic patients develop bulimia and vice versa [8].

The college population is subject to several sociological and cultural changes. Many students move from the family nucleus and become responsible for their own eating habits, organizing their time, buying food, preparing their menus, and organizing meal schedules. All this can lead to skipping meals on a regular basis, preferring fast food, consuming alcohol, smoking, and finally favoring the appearance of EDs [9,10].

Academic stress can be associated with bulimic behaviors, as risk behaviors or thoughts can be generated as a factor in combating stress, increasing the possibility of developing some of the eating disorders [7].

A meta-analysis in 2009 found that weight gain in the first year of college is 2 kg; however, it should be borne in mind that the increase in weight of 2 kg in just 8 months is higher than that in the rest of the population; therefore, thorough study of what is happening is required because this will mark obesity in adulthood [11].

A cross-sectional study was conducted among students at the University of Rouen-Normandy, France. A total of 1943 college students, with a mean age of 20.1 (±1.9) were evaluated. In the results, 63.4% were woman, 8.6% underweight, 12.4% overweight, and 4.6% obese, with a prevalence of EDs of 24.8%, especially in female students (31.6%) compared with males (17.0%) (*p* < 0.001), being bulimic 13.3%, hyperphagic 8.6% and restrictive 2.9% [12]. In Ontario (Canada), they studied 301 students aged 17 to 20 years from Brock University during the first year of college and demonstrated that male and female students, and especially male, undergo changes in nutrition and body weight/composition, making their diet worse, increasing body weight, BMI, waist-to-hip ratio, and body fat, [10].

This phenomenon, related to weight gain during the first year of college, is known in English-speaking countries as “Freshman 15”. This refers to students gaining an average of 15 lbs. (6.8 kg) during this first year of college. However, the latest studies show that weight gain is not as high as indicated by this age. Being away from home can favor a weight gain from 0.68 kg to 4 kg; the cause is still unclear, and no relationship has been seen between gender [11,13].

One of the few studies carried out in Spain was carried out at the University of Extremadura, where 600 students were studied between the ages of 18 and 26. It was observed that the students had a few adherences to the Mediterranean diet (MD) using the Mediterranean Diet Adherence Test (MEDAS-14) [14,15]. After the descriptive analysis, they obtained similar averages in the group of girls and boys (5.78 for the boys and 5.69 for the girls).

Another study studied 597 university students between 17 and 20 years old, from southern Spain, on adherence to the MD using the KIDMED test [16], where 77.6% had an optimal diet, while 21.9% needed to improve their diet [17].

It is vitally important that during the university period, students will adopt healthy behaviors to control multiple risk factors that influence the future lives of young adults. They need to take care of themselves and be responsible for their food habits and lifestyles [17].

In addition, the situation of hygiene emergency linked to the pandemic of COVID-19 has caused the emergence of problems with alimentary consumption that can heighten during this first year of university [18]. Additionally, in this last period of time and due to regional confinements, many students have undertaken more visits to their relatives and virtual teaching has increased social distancing between students. In a study carried out during the first lockdown caused by COVID-19, students with culinary skills survived better and acknowledged having had a better diet than students without these skills [19].

To all this must be added the insecurity, in general, that withdrawing from the family environment entails, which adds to insecurity in decision-making related to food and also conditions eating behavior during the first year of university [20].

The main objective of this study is:

- Determine the eating behavior and predisposition to suffer EDs in first-year college students.

The secondary objectives are:

- Determine the prevalence of eating-disorder risk in first-year college students, through validated test of EDs.
- Determine the occurrence of changes in eating habits during the first year of college.
- Appraise changes in the weight, waist circumference and triceps fold of the participating individuals.
- Determine the emotional intake of participants.
- Assess the degree of adherence to the Mediterranean Diet.
- Rate eating behavior, including eating disorders during the COVID-19 pandemic.

## 2. Materials and Methods

### 2.1. Study Design

A multicenter prospective observational study that evaluates the predisposition of first-year university students to suffer from EDs through different validated tests as well as a record of different anthropometric measures, a record of intake, assessment of emotional intake and the degree of adherence to the Mediterranean Diet. This protocol of investigation follows the STROBE guides for transversal studies (Strengthening the Reporting of Observational Studies in Epidemiology).

This project will be carried out jointly by the Universitat de Vic-Universitat Central de Catalunya (Barcelona, Spain) with the Universidad Europea del Atlántico (Santander, Spain) and will monitor first-year students residing in university residences, student flats and family residences in the cities of Vic and Santander.

The project will be carried out in three phases that can be observed in Table 1.

The recruitment interview that includes the explanation of the project, the suitability of the participant based on the inclusion and exclusion criteria, anthropometric measurements and bioimpedance will be carried out in the simulation laboratories of Universitat de Vic-Universitat Central de Catalunya and in the University Clinic of the Universidad Europea del Atlántico.

### 2.2. Participant Recruitment

Study participants will be recruited in the different university residences with prior authorization by the residences, and on the various university campuses with the corresponding authorization.

An email will be sent through the virtual campuses of the two universities to all students enrolled in the first year, inviting them to participate in this study. In this email, the volunteers will be offered contact with the main investigator of the study, may request more information and may arrange an initial interview to assess whether this volunteer can be included in the study, according to the inclusion and exclusion criteria set out in Table 2.

Participants who are not excluded will be given the informed consent document; it will be explained again how the study will work and what is expected of them. When they sign the document, they will be included in the project, and their participation will begin.

Each participant will be identified with a random and individual code that they will need to use to identify the different documents that have to be filled out.

### 2.3. Data Collection and Measures

The valid and reliable measurement methods used to answer the study’s research question are listed in Table 1 and explained below:

1. Online survey, the link, and an individual code will be provided via email. Through this first contact with the student, a set of sociodemographic data (gender, date of birth, origin, email address, place of residence and current academic studies) and a series of parameters related to their lifestyle will be collected: physical activity, eating habits and habits related to their food intake. The following survey presents the various screening tests that the student must complete:

The EAT-26 is a globally used self-administered questionnaire, the summary version of which has properties of confidence and validity for EDs screening [26]. It was designed with the purpose of developing and validating a rating scale that would be useful in assessing a wide range of behaviors and attitudes present in AN. It initially had 40 items (EAT-40), but studies regarding its psychometric qualities led to an abbreviation of the same, resulting in a 26-item questionnaire, which was validated in a Spanish population in 2010 [21]. The instrument consists of three subscales: (a) diet: includes 13 items on avoidant behaviors in regard to fattening foods and concerns about thinness; (b) bulimia and food concern: includes six items on bulimic behaviors and thoughts related to food, and (c) oral control: includes the last seven items on self-control of intake and external pressure for weight gain. Each question has six answer options with different scores: 0 points (never, rarely, sometimes); 1 point (often); 2 points (very often); and 3 points (always). The total score is the sum of the answers to the 26 items, considering that question 25 scores in reverse. The higher the score, the higher the risk of AN or BN. The optimal cut-off points for EAT-26 as a EDs screening questionnaire is 20 (sensitivity = 100%; specificity = 97.8%); therefore, if the final score is ≤20 points, there is no presence of EDs, and if the final score is >20 points, more research with a professional is required because an EDs could be presented.

The Teen Figure Drawing Scale [22] is a questionnaire that analyzes satisfaction with body image through two variables: perceived silhouette and desired silhouette. This test consists of seven human figures (silhouettes), which gradually increase in size, from an “extremely thin” figure on the left side to an “extremely robust” figure on the right side. Each figure is assigned a certain BMI, classified into (1) underweight, (2) low moderate weight, (3) low light weight, (4) normal weight, (5) overweight, (6) moderate overweight and (7) obesity. Participants choose a figure to describe themselves (real self) and another that they would like to resemble (ideal self), and then body dissatisfaction is conceptualized as a discrepancy between the two responses, i.e., a gap between the ideal self and the real self. Positive and negative signs indicate dissatisfaction with the body: the desire to get bigger or thinner.

The SCOFF survey is a short and simple instrument that has demonstrated adequate sensitivity and specificity for EDs screening [23]. This is a five-item scale that quantifies some of the central symptoms related to eating disorders over the past three months. It presents a dichotomous response pattern (yes/no). The affirmative answers receive a point so that total scores between 0 and 5 can be obtained. The questionnaire is positive, and there is a risk of presenting/displaying an EDs when the person answers affirmatively to two or more questions.

The BITE test [24] assesses the presence and severity of bulimic symptoms and the cognitive and emotional signs and symptoms associated with binges. It consists of 33 items divided into two different subscales: a symptom subscale with 30 items with a dichotomous response pattern (yes/no) and a severity subscale with five or seven response options depending on the item. The total score is the sum of the answers of the 33 items and is interpreted as follows: if the final score is <10 points (there is no problem in eating behavior); if the final score is between 10–20 points (presence of abnormal eating patterns); if the final score is >20 points (presence of abnormal feeding patterns with the possibility of presenting BN).

The MEDAS-14 [15] is a validated dietary assessment tool used in the PREDIMED study. It consists of 14 items and allows you to assess the degree of adherence to the pattern of the Mediterranean Diet (MD). Each question has two answer options with different scores. The total score is the sum of the answers of the 14 items and is interpreted as follows: if the final score is >8 points (good adherence to the MD); if the final score is between 5 and 8 points (adherence can be improved to the MD); and if the final score is <5 points (low adherence to MD).

The EEQ [25] was created with the aim of evaluating the effect of emotions on eating behavior. It consists of 10 items. Each question has four answer options with different scores: 0 points (never); 1 point (sometimes); 2 points (usually); and 3 points (always). The total score is the sum of the answers of the 10 items. For clinical practice, subjects are classified as follows: if the final score is between 0–5 points: Non-Emotional eater (person with great stability in terms of eating behavior, eats when feeling physiological hunger without taking into account external factors or emotions); if the final score is between 6–10 points: Low emotional Eater (non-emotional person with respect to their diet, except that certain foods influence their will and that food is more than just eating); if the final score is between 11–20 points: Emotional Eater (person who eats conditioned by their emotions, however, the food does not control their actions, so they maintain a certain mastery of the their food); if the final score is between 21–30 points: Very Emotional eater (person especially vulnerable to suffering from some kind of eating disorder (anorexia, bulimia) if no corrective measures are applied).

2. 72 hDR. Document to fill out—the 72 hDR is delivered after the first interview and after the appointment to take the anthropometric measurements of the 1st phase of the project. The first record must be returned to the team within the following 15 days, and the second must be delivered 15 days after taking anthropometric measurements of the 3rd phase. The participant must write down for three days (two weekdays and one weekend day) all the meals they prepare, the food they eat (inside and outside the main meals), the culinary techniques used and the quantities (in weights or homemade measures). The participant must return the document via email after fifteen days, in Word or PDF format. Each record will be entered into the ODIMET online diet calculation program that will provide us with the intake data at energy level (KJ/kcal), carbohydrates (g), proteins (g), total fats (g), saturated fatty acids (SFA) (g), monounsaturated fatty acids (MUFA) (g), polyunsaturated fatty acids (PUFA) (g) and cholesterol (g), fiber (g) vitamins and minerals.

3. Anthropometric measurements. Specific anthropometric measurements will be taken for each participant in each of the different phases: body weight (kg), body height (cm) and abdominal perimeter (cm) tricipital fold (mm) and bioimpedance (TANITA TBF300).

With regards to data collection and the consequent analysis of quantitative variables, the different answers obtained in the different phases of the project (initial, intermediate and final) will be compared for the same question.

### 2.4. Statistical Analysis

The sample size is calculated for finite populations by counting the students who enter the first year at both universities. It is calculated with a 95% confidence level, 5% of error range, a precision level (d = 3%) and an expected proportion of losses of 15%.

The universe of students of the first year for the Universitat de Vic-Universitat Central de Catalunya of (*n* = 2000 students), and the representative size of (*n* = 275 students). In the case of the Universidad Europea del Atlántico, the universe of (*n* = 400 students), and the representative sample size of (*n* = 168 students).

The analysis of the data will be performed as follows:

1. Normality distribution for quantitative variables will be assessed using the Kolmogorov–Smirnov normality test. The quantitative variables will be expressed as the mean and standard deviation, and the body mass index (BMI) will be distributed using WHO range classification.
2. For the dichotomic quantitative variables, Student’s *t*-test or the nonparametric Mann–Whitney test will be used and for the polycotomic ANOVA test, or the nonparametric Kruskal–Wallis test. Univariate comparisons will be investigated between groups extracted from the same tests, but from two groups (students living with parents and those living independently in residences or apartment flat). The fact of comparing means implies the fact of carrying out the Levene Test, which is undertaken to observe the equality or inequality of the variances in the two groups.
3. For the qualitative variables, we will make a contingency table to observe from each possible answer how many people have selected it. We will use the percentages in these variables and for the comparison of the data using the Pearson X2 test or nonparametric Fisher’s exact test for categorical data.
4. The criterion of significance is established at *p* < 0.05. All computer data will be analyzed using the “Statistical Package for the Social Sciences” (SPSS) software, version 26.0 for Windows.

### 2.5. Ethical Approval, Ethical Considerations and Dissemination

Data relating to study participants will be treated confidentially, always protecting the rights of participants. The use of these data is restricted to the research team, and its exploitation will be exclusive to the study. All study participants will do so voluntarily, and at any time during the study, they may decide to leave the same. Informed consent will be collected, and it will be verified that all patients have understood the signed documents.

All participants will be assigned a code so that the answers they give will not be linked directly to them, as a guarantee of confidentiality. The data obtained from this participation will not be used for any purpose other than that explicit in this research. They will be kept securely under the direct responsibility of the principal investigator.

Data protection will be followed by Organic Law 15/1999, of 13 December, on the protection of personal data and General Regulation (EU) 2016/679, of 27 April 2016, on data protection (RGPD).

The protocol was approved by the Ethics Committee of the University of Vic-Central University of Catalonia (57/2018) and by the ethics committee of the European University of the Atlantic (CEI-10/2018).

## 3. Discussion

The aim of this investigation is to identify the risk of alimentary behavior disorders in college students in the first year and to determine the alimentary habits and body composition of these individuals at two universities in Spain, and how the COVID-19 pandemic has affected the eating behavior of students.

College students are a vulnerable group in relation to their nutritional health, owing to changes in lifestyle and the production in young students of food insecurity and changes in body weight and composition [10,20,27].

On one hand, students increase the number of hours they are sitting at the university and in their own homes, and this increases their sedentary lifestyle. On the other hand, it presents a greater independence with regard to the hour they eat and the foods that they choose to consume, their eating schedule and the number of foods they eat throughout the day, even though some student’s become independent from the family home [28].

A healthy diet, such as the Mediterranean Diet, is one of the main warning factors of obesity or non-transmissible illnesses, such as diabetes mellitus type II [29]. A healthy diet would have to include a high consumption of fruit, vegetable and integral cereals and a low consumption of saturated fat, salt, and simple carbohydrates [30].

The college stage is a period of transition between adolescence and adulthood and, thereby, is a good moment to promote strategies of healthy eating [31].

The increase in the consumption of hypercaloric and unstable diets in this university stage shows poor adherence to the Mediterranean Diet of students [17].

The lack of time caused by an increase in the hours of study is one of the main barriers to healthy eating by university students, as well as the lack of sufficiently healthy foods in university canteens [31].

In addition, stress is another factor that influences changes in healthy habits, with students experiencing a greater degree of stress tending to consume less healthy junk foods [32].

Various studies have observed that the consumption of fruits and vegetables has declined significantly in the Dutch population [33] as in the Spanish population. In addition, university students in the Mediterranean area move away from the MD pattern, towards a diet high in saturated fat and simple sugars [34]. Moreno-Gómez et al. also observed, in 2012, that another problem was linked to the increase in alcohol consumption (up to 80% of respondents) and tobacco use (up to 35.9% of respondents) [34].

Negative emotions that have been studied and related to eating behavior include sadness, anger, frustration, anxiety, fear, and boredom. First-year college students experiencing negative emotions may be less motivated to make healthy food choices and more likely to make poor food choices [35].

Another notable change in the lives of the students will be the coexistence with other students who arrive at the university in this period 2020/21; In these cases, living without a family environment in a university environment may cause alterations in eating habits and facilitate the appearance of eating disorders. It's more, a post-pandemic experience of confinement, social distancing and the perception of the risk for their health due to COVID-19, can favor this type of pathology [19,20,36].

All these circumstances determine the need to study alimentary habits and the risk of suffering EDs in this community, and to be able to predict the usual prevalence of EDs, as well as the perception of the corporal image, real consumption, and alterations in different anthropometric parameters, to establish strategies of suitable alimentary education for the needs of young students in universities.

However, little up-to-date literature exists in Spain; neither is available research linked to the situation of the pandemic, which represents an opportunity for further research to be included in the analysis; additionally, the possible behaviors linked to consumption, or the follow-up of the MD, could provide answers to the needs of university students in this vital time of change and tied to the pandemic.

Although this study is a good opportunity for investigation, it is necessary to present the potential biases associated with the methodological design of this protocol. The possible biases include: capacity of recruitment of volunteers, of time in the reply of the surveys, of veracity in the self-reporting. To increase participation, the dissemination channels of the two universities will be used to recruit the necessary students for the study and presentations of the study will be made in the groups of first-year students to facilitate knowledge of the project and desire to participate. To guarantee the veracity of the data, information will be added on how to fill in the different tests and how to fill in the 72-h record. Moreover, the principal limitation would be the maintenance of the sample over time. We would expect that participation in both centers would reduce the risks and affect the results. In order not to lose participants, automatic reminders of the tasks to be carried out in the different phases will be sent. The study is planned to be carried out during two or three academic years in order to obtain the calculated sample.

## Figures and Tables

**Table 1:** Phases of the project.

Phase of Study	Period	Information Will Be Collected:
1st phase: Initial interview	September/October	An online survey will collect a series of parameters related to their lifestyle, physical activity, eating habits and habits related to their intake. And these tests: - The Eating Attitudes Test (EAT-26) [21]. - Teen Figure Drawings Scale [22]. - Eating Behavior Test (SCOFF) [23]. - Bulimia Investigatory Test Edinburgh (BITE) [24]. - MEDAS-14 [15]. - Emotional Eater Questionnaire (EEQ) [25]. - Appearances related with the consumption, the weight and the COVID-19. 72 h dietary record (72 hDR). Anthropometric measurements: weight, height, waist circumference, triceps fold and bioimpedance.
2nd phase: Intermediate interview	February	- Anthropometric measurements: weight, waist circumference, triceps fold and bioimpedance.
3rd phase: Final interview	April/May	An online survey will collect a series of parameters related to their lifestyle, physical activity, eating habits and habits related to their intake. And these tests: - EAT-26 [21]. - Teen Figure Drawings Scale [22]. - SCOFF [23]. - BITE [24]. - MEDAS-14 [15]. - EEQ [25]. 72 hDR. Anthropometric measurements: weight, waist circumference, triceps fold and bioimpedance.

**Table 2:** Inclusion and exclusion criteria.

Inclusion Criteria	Adult students (≥18 years). First-year students of different genders. First-year students residing in the university residences. First-year students residing in student flats in the city of Vic or Santander. First-year students residing in the family home.
Exclusion Criteria	Students residing in the university residences in the cities of Vic or Santander who take courses other than first-year. Students with a history or current diagnosis of EDs. Students with temporary residence type Erasmus. Students who do not commit to follow-up throughout the project. Students who have not signed the Informed Consent document.
